# Supplementary figures and images for: Dabrafenib, idelalisib and nintedanib act as significant allosteric modulator for dengue NS3 protease
Source: PLoS One. 2021 Sep 10;16(9):e0257206. doi: 10.1371/journal.pone.0257206 (PMC8432871; doi:10.1371/journal.pone.0257206)

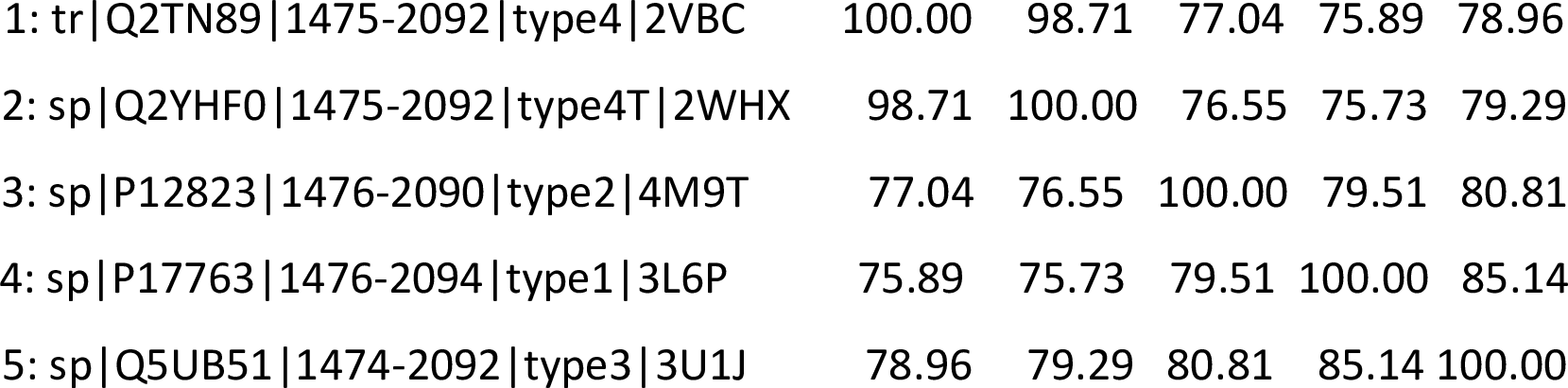

Supplement: S1 Fig — (TIF) [file pone.0257206.s001.tif]

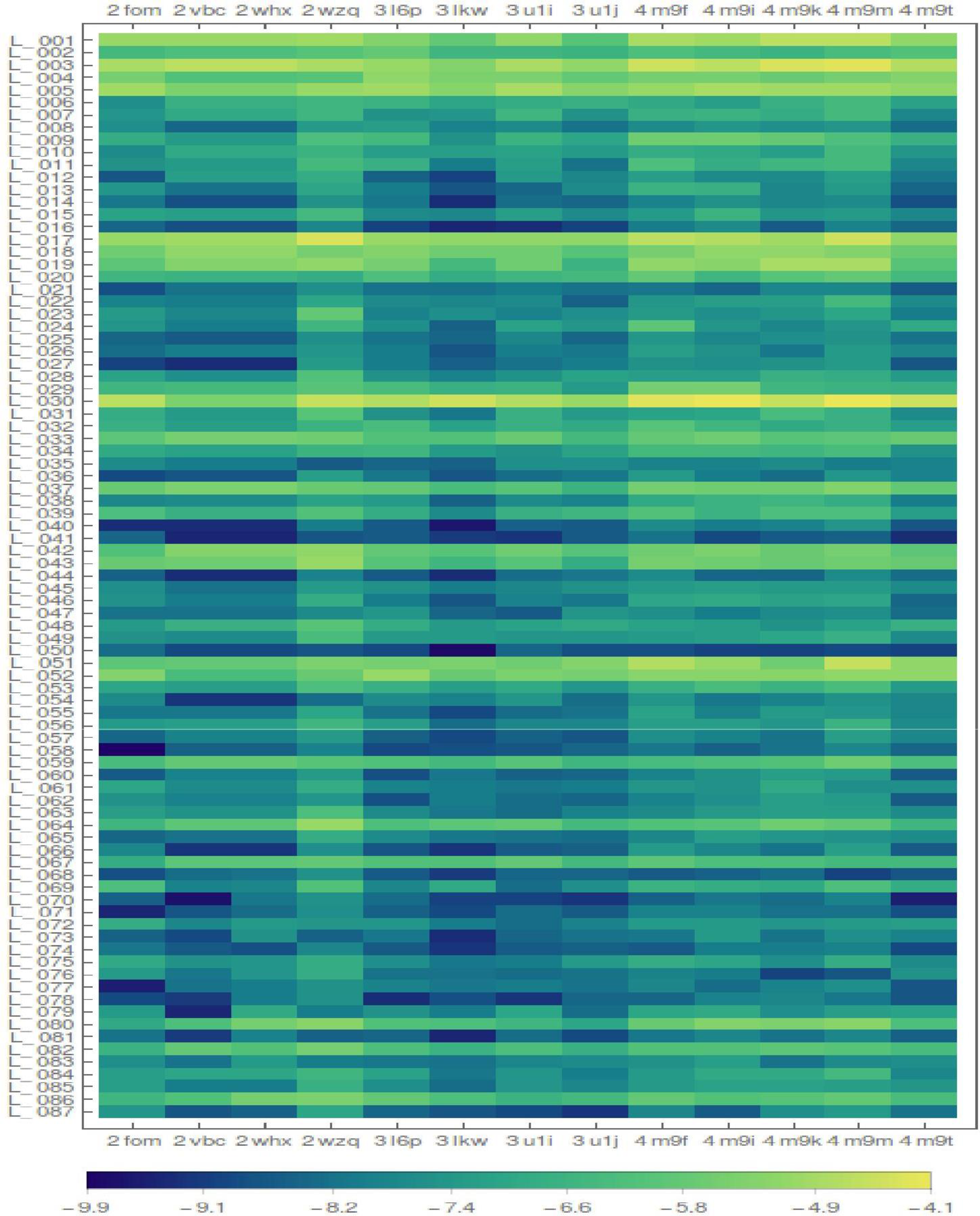

Supplement: S2 Fig — (TIF) [file pone.0257206.s002.tif]

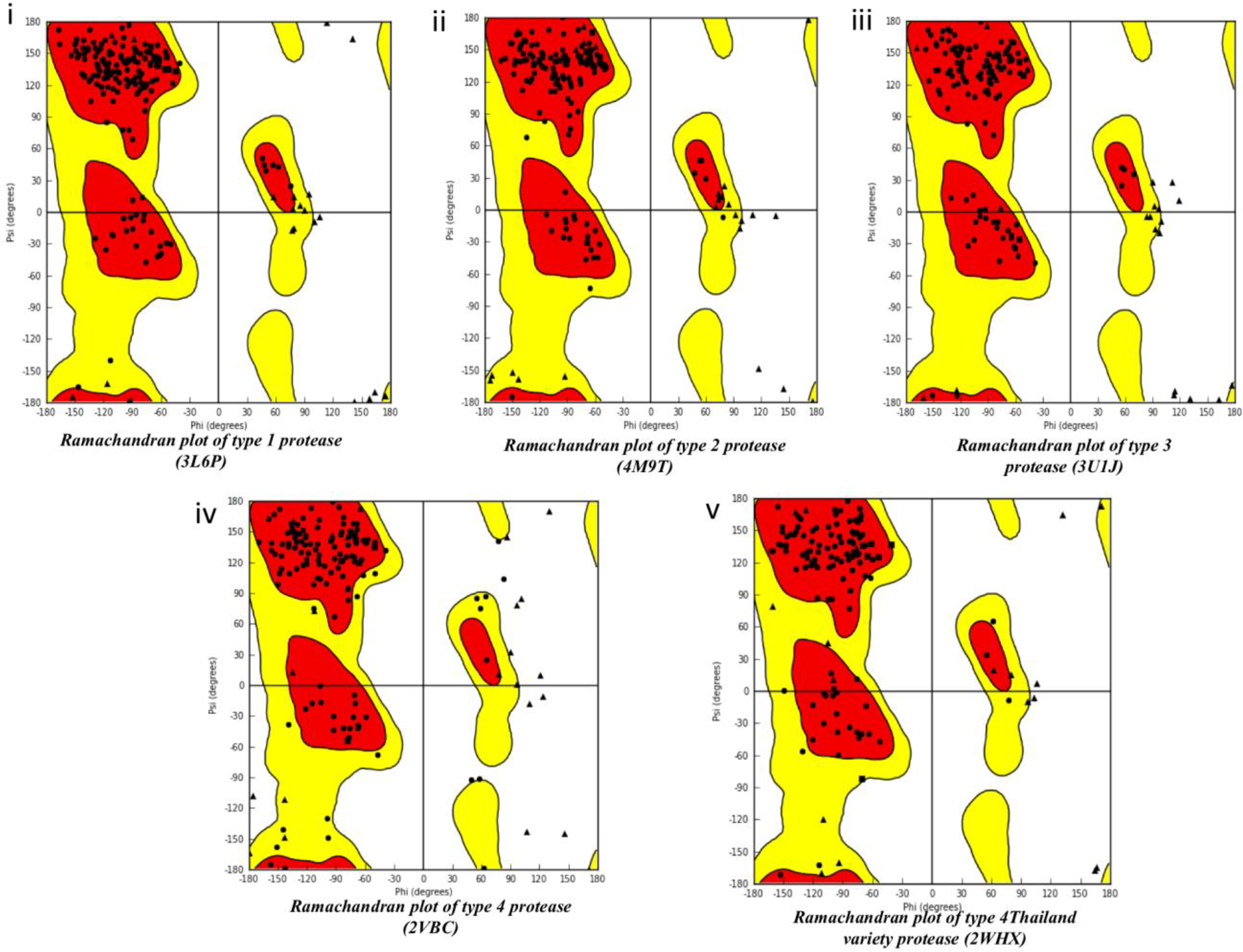

Supplement: S3 Fig — (TIF) [file pone.0257206.s003.tif]

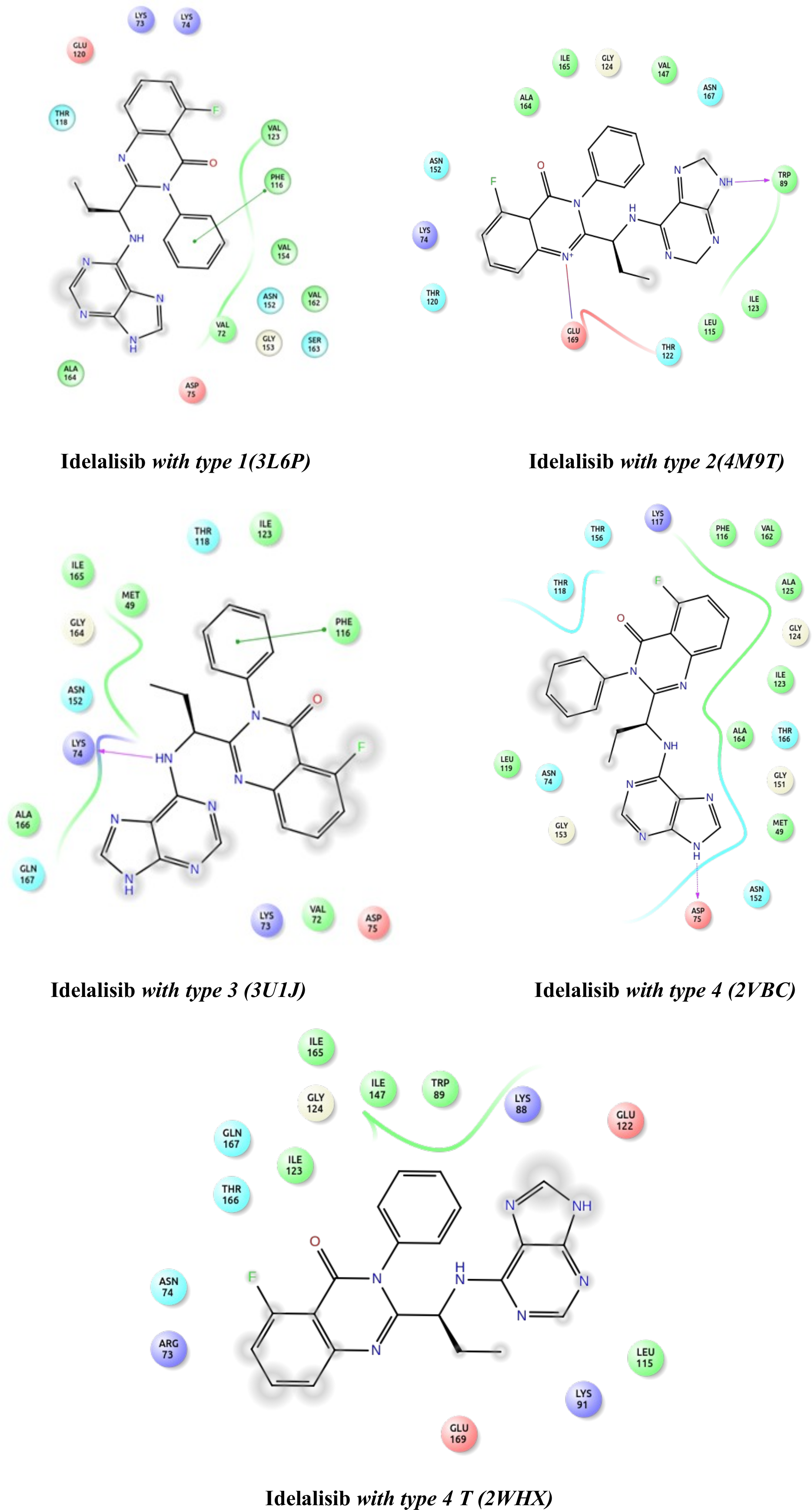

Supplement: S4 Fig — (TIFF) [file pone.0257206.s004.tiff]

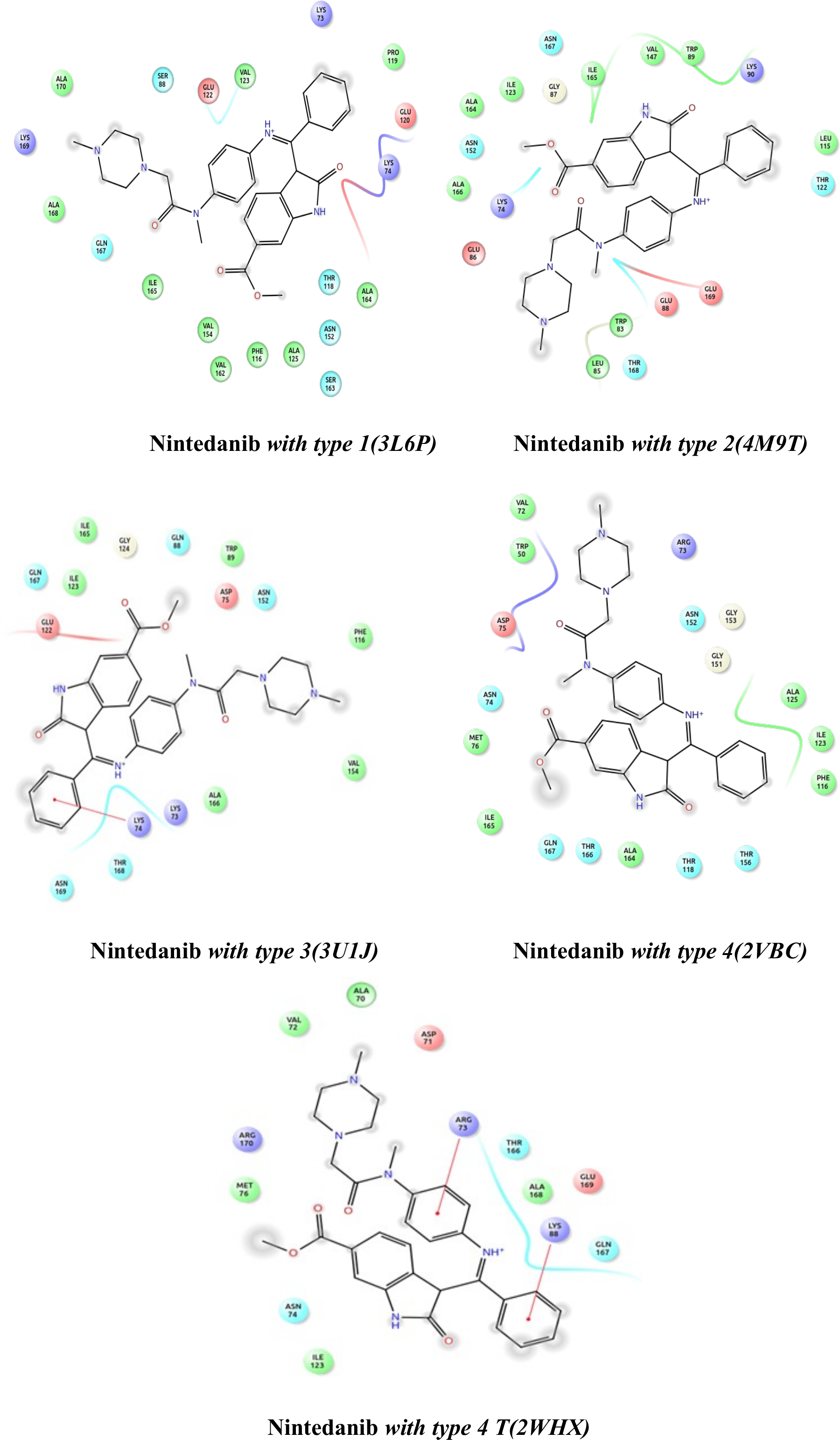

Supplement: S5 Fig — (TIFF) [file pone.0257206.s005.tiff]

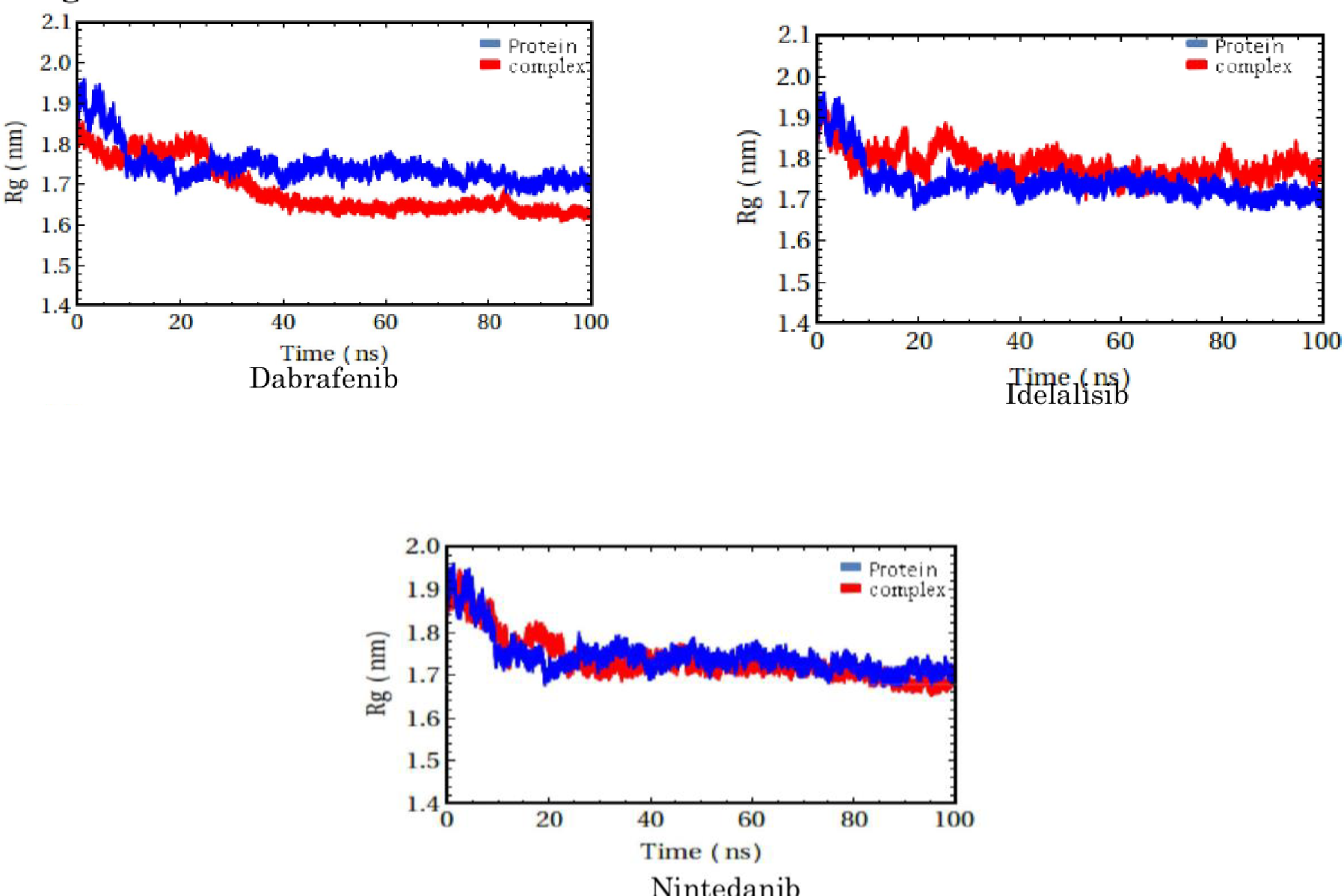

Supplement: S6 Fig — (TIF) [file pone.0257206.s006.tif]

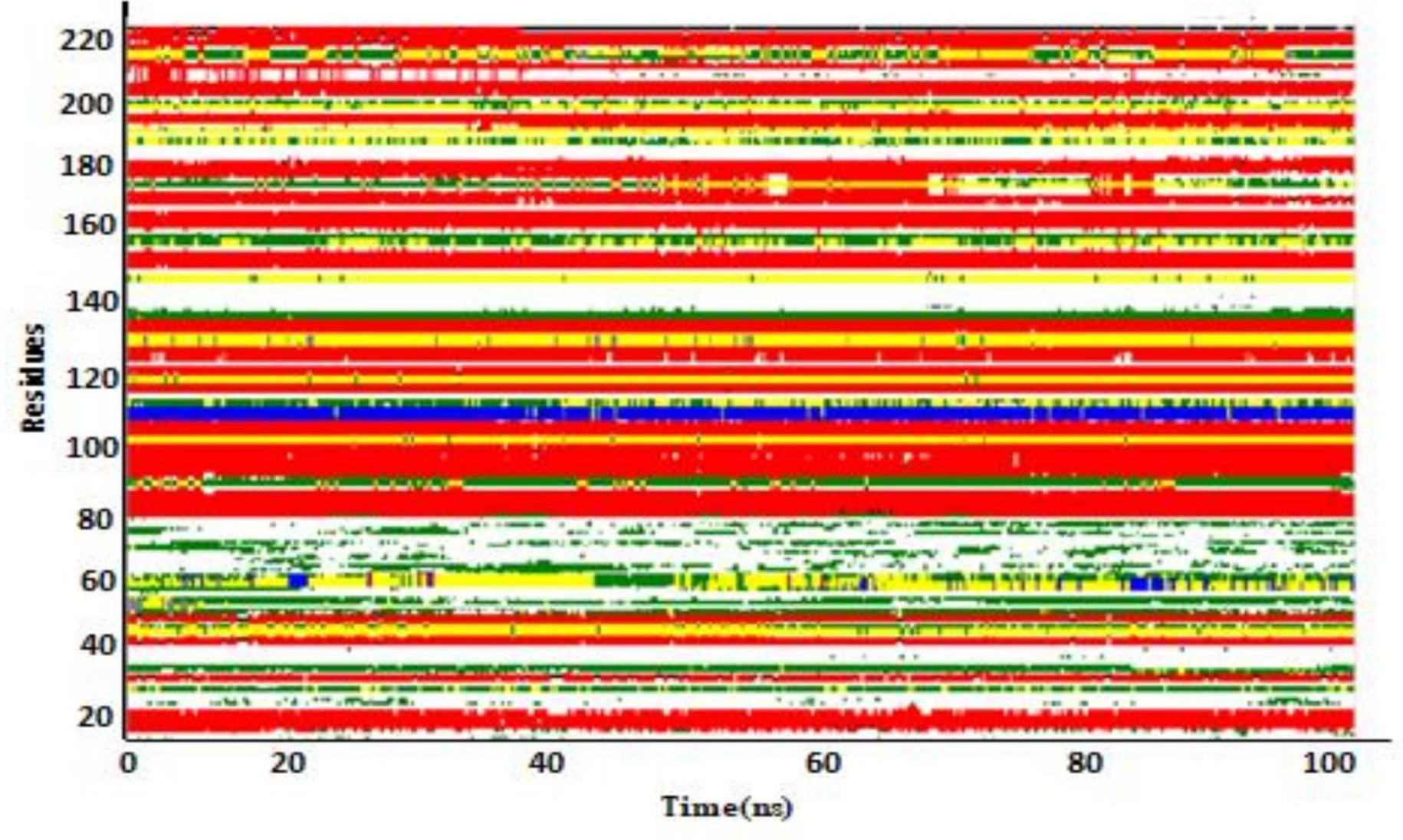

Supplement: S7 Fig — Color key: white, coil; red, beta-strand; black, beta-bridge; green, bend; yellow, turn; blue, alpha-helix; purple, pi-helix; gray, 3–10 helix. (TIF) [file pone.0257206.s007.tif]

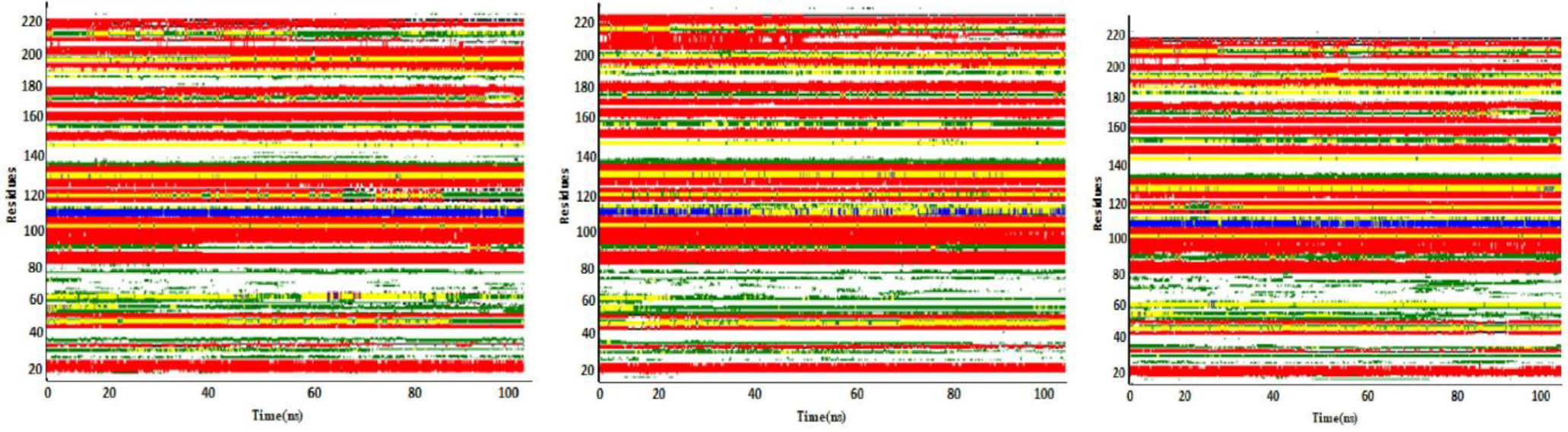

Supplement: S8 Fig — Color key: white, coil; red, beta-strand; black, beta-bridge; green, bend; yellow, turn; blue, alpha-helix; purple, pi-helix; gray, 3–10 helix. (TIF) [file pone.0257206.s008.tif]
