# Supplementary material for: Dabrafenib, idelalisib and nintedanib act as significant allosteric modulator for dengue NS3 protease
Source: PLoS One. 2021 Sep 10;16(9):e0257206. doi: 10.1371/journal.pone.0257206 (PMC8432871; doi:10.1371/journal.pone.0257206)
Supplement: S1 Table — (DOCX) [file pone.0257206.s009.docx]

**S1 Table**: List of the drug molecules along with their accession codes.

| Sl No. | Name |
| --- | --- |
| L_001 | 1-Deoxynojirimycin |
| L_002 | 3,4-Dichloroisocoumarin |
| L_003 | 3-Oxo-2,3-dihydro-1,2-oxazole-4-carboxylic acid |
| L_004 | 4-(2-Aminoethyl)benzenesulfonyl fluoride hydrochloride |
| L_005 | 4-Aminobenzamidine |
| L_006 | Abacavir |
| L_007 | Adp |
| L_008 | Afatinib |
| L_009 | Aliskiren |
| L_010 | AMPPNP |
| L_011 | Aprotinin, bovine |
| L_012 | Argatroban 1 |
| L_013 | Argatroban 2 |
| L_014 | Axitinib |
| L_015 | Bosutinib |
| L_016 | Cabozantinib |
| L_017 | Captopril |
| L_018 | (-)-Castanospermine |
| L_019 | (+)-Castanospermine |
| L_020 | Cetuximab |
| L_021 | Cobimitinib |
| L_022 | Comp6-1 |
| L_023 | Comp6-2 |
| L_024 | Comp6-3 |
| L_025 | Crizotinib |
| L_026 | Dabigatran |
| L_027 | Dasatinib |
| L_028 | Desmethylclozapine |
| L_029 | Didanosine |
| L_030 | Diisopropylfluorophosphate |
| L_031 | DTNB |
| L_032 | Efavirenz |
| L_033 | Emtricitabine |
| L_034 | Erlotinib |
| L_035 | Etravirine |
| L_036 | Fostamatinib |
| L_037 | Fucoidan |
| L_038 | Gefitinib |
| L_039 | Glucan |
| L_040 | Ibrutinib |
| L_041 | Imatinib |
| L_042 | (-)-Lamivudine |
| L_043 | (+)-Lamivudine |
| L_044 | Lapatinib |
| L_045 | Lenvatinib |
| L_046 | Minocycline |
| L_047 | Mubritinib |
| L_048 | Nevirapine |
| L_049 | Norclozapine |
| L_050 | Pazopanib |
| L_051 | Pegaptanib |
| L_052 | Phenylmethanesulfonyl fluoride |
| L_053 | prochlorperazine |
| L_054 | Rilpivirine |
| L_055 | Rivaroxaban |
| L_056 | Ruxolitinib |
| L_057 | Sitagliptin |
| L_058 | Sorafenib |
| L_059 | stavudine |
| L_060 | SU6656 1 |
| L_061 | SU6656 2 |
| L_062 | SU6656 3 |
| L_063 | Sunitinib |
| L_064 | Tenofovir |
| L_065 | Vandetanib |
| L_066 | Vemurafenib |
| L_067 | Zidovudine |
| L_068 | Alectinib |
| L_069 | Atazanavir |
| L_070 | Dabrafenib |
| L_071 | Daclatasvir |
| L_072 | Darunavir |
| L_073 | Dasabuvir |
| L_074 | Dolutegravir |
| L_075 | Elvitegravir |
| L_076 | Grazoprevir |
| L_077 | Idelalisib |
| L_078 | Nintedanib |
| L_079 | Ombitasvir |
| L_080 | Peramivir |
| L_081 | Raltegravir |
| L_082 | Ribavirin |
| L_083 | Ritonavir |
| L_084 | Ruxolitinib |
| L_085 | Sofosbuvir |
| L_086 | Tenofovir |
| L_087 | Trametinib |
