# Supplementary material for: Dabrafenib, idelalisib and nintedanib act as significant allosteric modulator for dengue NS3 protease
Source: PLoS One. 2021 Sep 10;16(9):e0257206. doi: 10.1371/journal.pone.0257206 (PMC8432871; doi:10.1371/journal.pone.0257206)
Supplement: S2 Table — PDB IDs marked with * are the 3D structures containing protease domain. Note:—1,2,3,4, refers to DENV types and 4T refers to DENV 4 Thailand variety. (DOCX) [file pone.0257206.s010.docx]

**S2 Table**: Available crystal structures of DENV NS3 protein. PDB IDs marked with * are the 3D structures containing protease domain.

| **Sl.** | **PDB ID** | **UNIPROT ID** | **Length** | **Resolution (Å)** | **Serotype** |
| --- | --- | --- | --- | --- | --- |
| 1 | 4M9I* | Q91H74 | 247 | 2.4 | 2 |
| 2 | 4M9K* | Q91H74 | 247 | 1.46 | 2 |
| 3 | 4M9M* | Q91H74 | 247 | 1.53 | 2 |
| 4 | 3U1I* | Q5UB51 | 494 | 2.3 | 3 |
| 5 | 3U1J* | Q5UB51 | 191 | 1.8 | 3 |
| 6 | 2WHX* | Q5UB51 | 618 | 2.2 | 4T |
| 7 | 2WZQ* | Q2YHF0 | 619 | 2.8 | 4T |
| 8 | 3L6P* | P17763 | 236 | 2.2 | 1 |
| 9 | 3LKW* | P17763 | 236 | 2 | 1 |
| 10 | 2JLQ | Q2YHF0 | 451 | 1.67 | 4T |
| 11 | 2JLR | Q2YHF0 | 451 | 2 | 4T |
| 12 | 2JLS | Q2YHF0 | 451 | 2.23 | 4T |
| 13 | 2JLU | Q2YHF0 | 451 | 2.04 | 4T |
| 14 | 2JLV | Q2YHF0 | 451 | 1.9 | 4T |
| 15 | 2JLW | Q2YHF0 | 451 | 2.6 | 4T |
| 16 | 2JLX | Q2YHF0 | 451 | 2.2 | 4T |
| 17 | 2JLY | Q2YHF0 | 451 | 2.4 | 4T |
| 18 | 2JLZ | Q2YHF0 | 451 | 2.2 | 4T |
| 19 | 2VBC* | Q2TN89 | 618 | 3.15 | 4 |
| 20 | 2FOM* | Q91H74 | 185 | 1.5 | 2 |
| 21 | 2BMF | Q91H74 | 451 | 2.41 | 2 |
| 22 | 2BHR | Q91H74 | 451 | 2.8 | 2 |
| 23 | 4M9F* | Q91H74 | 247 | 2.7 | 2 |
| 24 | 4M9T* | Q91H74 | 247 | 1.74 | 2 |
